# The Public Knowledge of Precision Medicine and Genomic Research: A Survey in the Aosta Valley

**DOI:** 10.3390/jpm15030080

**Published:** 2025-02-24

**Authors:** Matteo Mongelli, Biagio De Angelis, Valeria delle Cave, Giuliano Greco, Arianna De Arcangelis, Andrea Bernagozzi, Chiara Salvemini, Matteo Calabrese, Jean Marc Christille, Andrea Cavalli, Stefano Gustincich, Maria Grazia Monaci

**Affiliations:** 1 CMP3VdA, Istituto Italiano di Tecnologia (IIT), Via Lavoratori Vittime del Col du Mont, 28, 11100 Aosta, Italy; matteo.mongelli@iit.it (M.M.); biagio.deangelis@iit.it (B.D.A.); arianna.dearcangelis@iit.it (A.D.A.); bernagozzi@oavda.it (A.B.); salvemini@oavda.it (C.S.); calabrese@oavda.it (M.C.); direttore@oavda.it (J.M.C.); andrea.cavalli@iit.it (A.C.); stefano.gustincich@iit.it (S.G.); 2 Communication and External Relations Directorate, Istituto Italiano di Tecnologia (IIT), Via Morego 30, 16163 Genova, Italygiuliano.greco@iit.it (G.G.); 3 Fondazione Clément Fillietroz, Astronomical Observatory of the Autonomous Region of the Aosta Valley (OAVdA), Loc. Lignan 39, 11020 Nus, Italy; 4 Computational and Chemical Biology, Istituto Italiano di Tecnologia (IIT), Via Morego 30, 16163 Genova, Italy; 5 Center for Human Technologies, Non-Coding RNAs and RNA-Based Therapeutics, Istituto Italiano di Tecnologia (IIT), Via Enrico Melen 83, 16152 Genova, Italy; 6 Department of Human and Social Science, University of Valle d’Aosta, Strada Cappuccini 2A, 11100 Aosta, Italy

**Keywords:** precision medicine, public health, survey, genomic medicine

## Abstract

**Background**: Precision medicine (PM) considers the genetic variability of individuals to identify tailored diagnosis and treatments. It relies on the possibility of gathering the widest possible health data and genetic information from individuals to obtain a broad pool of comparative data. To achieve this goal, the Region of Valle d’Aosta, since 2019, has co-financed the research center CMP^3^VdA, aiming to sequence 5000 genomes of patients with neurodevelopmental, neurodegenerative, oncological, and organ transplantation diseases, and to investigate the genetic variability of the resident population. **Methods:** This paper presents the results of an online survey of 472 (328F) respondents regarding willingness to participate in the genomic project and awareness, attitudes, and concerns about PM. **Results**: The main results show that the vast majority (92.6%) would be willing to participate—a higher percentage than in previous studies. Age, education, and prior experience in the healthcare sector are significant factors influencing the awareness of PM. Additionally, subgroups organized by age, gender, and religiosity show significant differences with respect to participants’ reasons for participating in research and which types of biological samples they would be willing to donate. **Conclusions**: Our findings can serve as a guide for stakeholders—particularly policymakers—to target institutional communication and achieve maximum participation in genomic research projects.

## 1. Introduction

The advancement in the field of omics sciences; the adoption of next-generation sequencing technologies, which have allowed for a reduction in sequencing costs [1,2,3]; the development of third-generation technologies; and the creation of massive datasets for genomics big data [4] have represented a step forward for the integration of precision medicine (PM) as a central element of healthcare science [5]. Today, PM holds significant relevance in various fields, such as cardiology [6], diabetology [7], nephrology [8], neurology [9,10,11], oncology [12,13], ophthalmology [14], and psychiatry [15,16].

To ensure the success of PM, it is essential to acquire high-quality clinical data and the ability to compare them with genomic data from a large number of individuals. The United States of America [17], the United Kingdom [18], Poland [19], Italy [20], Estonia [21], and other countries are investing in PM programs, aiming at improving population health outcomes.

Although various studies on the population’s perception of PM have been conducted in different countries, including Switzerland [22,23,24], Poland [25], Singapore [26], the Republic of Korea [27], and Japan [28], not much has been written about public opinion in Italy. In 2018, a survey was carried out among 1113 healthcare professionals, showing that about 1 in 2 of them were not sufficiently informed about PM, and 40.7% claimed that they were just partially informed about it [29]. A subsequent study was conducted among 145 family members who were assisting patients attending a geriatric or neurological visit, and it investigated the willingness to provide biological samples to set up of a biobank for research purposes. The survey revealed that 86% of the subjects declared themselves to be ready to provide biological samples. This percentage was not significantly influenced by gender or age but was affected by educational level [30].

These studies outlined the frame of the perception of PM in Italy; however, they did not describe a general overview of the population’s thoughts since they were addressed to specific categories. Understanding what ordinary people think about this topic is important in supporting the efforts that regional governments are putting in place to promote personalized healthcare therapies and research. In the Aosta Valley region, the CMP^3^VdA research center and the 5000genomi@VdA project are underway. The goal of the project is to sequence 5000 genomes of patients with neurodevelopmental, neurodegenerative, oncological, and organ transplantation diseases and to investigate the genetic variability of the resident population.

Here, we report the results of a cross-sectional survey conducted on the inhabitants of the Aosta Valley region, focusing on willingness to participate in a genomic research project and on awareness of PM, exploring attitudes and concerns about genomic testing, willingness to provide biological samples, and the type of results that participants would be willing to receive.

## 2. Materials and Methods

### 2.1. Respondents

The survey was administered online through Google Forms between July and September 2023. The sample was recruited via the institutional e-mails of the partners participating in the 5000genomi@VdA project and of local authorities. Participation was voluntary, and respondents were assured that the data would be analyzed in an aggregate and anonymous manner for statistical research purposes. Participants were required to be over the age of 18 and were asked to provide informed consent. Data Protection Officers of all the involved partners verified whether data regulation rules were complied with. The study was conducted in accordance with the Declaration of Helsinki and approved by the Ethic Committee of the University of Valle d’Aosta (Prot. 0011487, 7 July 2023). The survey was designed to be easily completed in 8–10 min.

Respondents totaled 480 people; 8 did not provide consent; therefore, the final sample consists of 472 people (328F). Table 1 shows expanded demographics. Given the cross-sectional nature of our study, we aimed at collecting data on a large sample (i.e., N > 450) that would guarantee sufficient power to detect effects, with α = 0.05 and 1 − β = 0.90, via a moderate magnitude and sensitivity analysis, as determined by a priori power analysis conducted using G*Power [31]. The recruitment of respondents was discontinued once the requisite number had been obtained.

### 2.2. Questionnaire

The questionnaire was developed after a careful review of previous studies [23,27,28] conducted on public attitudes toward PM. The questionnaire included dichotomic Yes/No questions, multiple responses, and level of agreement with several statements using 5-point Likert scales (from 1 “Strongly disagree” to 5 “Strongly agree”) for a total of 46 questions in four sections. The final version—which is in Italian and is available in the Supplementary Materials—was reviewed by experts in genomics and pretested with 12 non-experts in the health sciences. A brief definition of precision medicine was provided to participants in the introduction. The first measures focus on awareness of PM and willingness to participate to a research project with genetic testing (both with a dichotomic Yes/No answer). The second question acted as a filter and directed participants to two sections concerning, respectively, motivation and concerns about participating/not participating in genomic research and willingness to provide health data and biological samples for personalized health research. A third section regarded data management, the sharing of the results, and data ownership and protection. Finally, sociodemographic and lifestyle health-related (smoking, alcohol consumption, health general status) information was collected.

### 2.3. Data Analysis

We analyzed the data using the SPSS 27.0 with a significance level of α = 0.05 (*p* < 0.05). All categorical variables were expressed as frequencies and percentages and compared in subgroups for sociodemographic and lifestyle categories using a chi-square test. For continuous variables, principal component analyses were first conducted on the sets of items regarding concerns about participating, or not, in a genomic research project and regarding data management and sharing to obtain dimension reductions and increasing interpretability but, at the same time, minimizing information loss and preserving as much variability as possible; secondly, the identified dimensions and other continuous variables were compared in subgroups with appropriate *t*-tests or one-way Anova testing. Prior to conducting the analysis, we assessed the normality assumption of our dependent variables using skewness and kurtosis measures. The results indicated a mild departure from normality for only one of the identified composite variables and for three out of six continuous behavioral variables related to willingness to provide biological samples. Although analysis of variance can perform poorly with non-normal data, this is primarily the case with small sample sizes. For non-normal data, a sufficiently large sample size is required, and our sample is large enough to support parametric testing, which yields results that are readable, easily interpretable, and comparable to other analyses conducted within our cross-sectional design, with the majority of variables falling within the normality assumption limits.

Therefore, our cross-sectional analyses considered the following variable: age, gender, education, having biological children, work experience in the healthcare sector, religiosity, health status, and lifestyle factors (alcohol consumption and smoking).

## 3. Results

Table 1 presents the respondents’ sociodemographic characteristics together with their Yes/No answers to questions of their willingness to participate in the research (participants were asked “Would you participate in a medical–scientific research project that uses your health data and/or biological samples?”) and their awareness of PM (“Have you ever heard of precision medicine?”).

A large majority (92.6%) of respondents reported a willingness to participate in the research project, with no significant differences in subgroups identified according to sociodemographic or lifestyle variables.

Regarding the awareness of PM, according to the subgroup chi-square comparisons, the youngest age group (18–24) was found to be significantly less aware compared to all older age groups. Level of education was also associated with awareness: compared to respondents with upper education (tertiary and post-university), those with compulsory and secondary education are less aware. Furthermore, respondents with previous work experience in the health sector were also found to be significantly more aware of PM.

### 3.1. Concerns About Participating

The 437 respondents who said they would participate in the genomic research project were asked to assess their motivations through nine Likert-scale questions. The three most important reasons were as follows: “To increase scientific knowledge” (M = 4.57; SD = 0.57), “To improve healthcare” (M = 4.52; SD = 0.59), and “To benefit society” (M = 5.52; SD = 0.62). These were followed by “To bring an advantage to my family” (M = 4.42; SD = 0.73), “To learn something about my health” (M = 4.34; SD = 0.67), “It is important for society” (M = 4.24; SD = 0.80), “It is important for my family” (M = 4.09; SD = 0.90), “It is important for myself” (M = 4.03; SD = 0.88), and “Out of a sense of duty” (M = 3.74; SD = 1.0). **A principal component analysis on these nine items resulted in an optimal two-factors solution after varimax rotation. These two components, both with eigenvalues greater than 1, accounted for 61% of the total variance. The first dimension comprised the five items related to benefits to society (“To increase scientific knowledge”, “To improve healthcare”, “To benefit society”, “Out of a sense of duty”, and “It is important for society”; Cronbach α = 0.80). The second dimension comprised the four items related to benefits to individual (“To bring an advantage to my family”, “To learn something about my health”, “It is important for my family”, and “It is important for myself”; α = 0.83). Two composite scores were obtained from the average of the items’ loading on the two components, both with excellent reliability, and subsequently. the means scores were compared in the respondents’ subgroups. In the one-way comparison, younger respondents (18–24) had a significantly lower score (M = 3.9; SD = 0.59) on the dimension “Benefits for Society” compared to all other age groups (F(5,439) = 4.9; *p* = 0.001). Considering education, respondents with only compulsory school education levels (i.e., primary and middle school) had higher scores (M = 4.6; SD = 0.48) compared to all other subgroups. Significant differences emerged also in the *t*-test for both dimensions, with higher mean scores in respondents having biological children (“Benefits to society” (M = 4.41, SD = 0.50 vs. M = 4.25, SD = 0.55; t(432) = 3.2; *p* = 0.002); “Benefits to individual” (M = 4.29, SD = 0.63 vs. M = 4.12, SD = 0.68; t(432) = 2.11, *p* = 0.036)).

### 3.2. Willingness to Provide Personal Information and Biological Samples

Respondents were asked about how much they would agree to give personal data for research, with five Likert scales. The three highest levels of agreement were for providing information about “Personal health” (M = 4.39; SD = 0.61), “Personal medical record” (M = 4.25; SD = 0.78), and “Family health history” (M = 4.16; SD = 0.83). There were followed by “Data from mobile digital applications” (step and counter calories, etc.) (M = 3.61; SD = 1.2), and lastly, by “Social media data” (M = 2.62; SD = 1.4). At the subgroup comparisons, significant differences emerged for age, with the younger group (18–24) being significantly less willing to give information about “Family health history” (F(1,434) = 3.59, *p* = 0.05) and “Social media data” (F(1,434) = 7.66, *p* = 0.006) compared to all older age groups. The difference between respondents with children was also found to be marginally significant, with higher scores (M = 4.23; SD = 0.78) vs. respondents without biological children (M = 4.08; SD = 0.86; t(429) = 1.8, *p* = 0.07) on the question of giving information about family health history.

A further question investigated the level of agreement about providing biological samples. Higher agreement was found with respect to providing saliva (M = 4.46; SD = 0.61), followed by urine (M = 4.43; SD = 0.62), hair (M= 4.40; SD = 0.72), blood (M = 4.39; SD = 0.69), feces (M = 4.28; SD = 0.85), and lastly, tissues (M = 4.01; SD = 0.99). In the subgroups comparisons, significant differences emerged for age, with the younger (18–24) group being less willing to provide blood (M = 4.12; SD = 0.80, F(1,435) = 7.28, *p* = 0.007) compared to older respondents (M = 4.42; SD = 0.67), and with the two younger groups (18–24/25–34) being less willing to provide feces (M = 3.95; SD = 1.02/M = 3.83; SD = 1.24; F(5,435) = 5.24, *p* = 0.001) and tissues (M = 3.50; SD = 1.22/M = 3.72; SD = 1.17; F(1,435) = 4.84, *p* = 0.001). On the willingness to provide biological samples, several significant differences also emerged for gender. On the question of whether women are less willing to provide tissues (M = 4.17; SD = 0.93) compared to men (M = 4.17, SD = 0.101; F(2,435) = 5.24, *p* = 0.001), the respondents who preferred not to say their gender or chose “other” were less willing to provide saliva (M = 4.08, SD = 1.19; F(2,435) = 2.89, *p* = 0.05) urine (M = 4.00, SD = 1.08; F(2,435) = 3.28, *p* = 0.038) and hair (M = 3.85, SD = 1.04; F(2,435) = 4.05, *p* = 0.018). Finally, individuals who declared themselves religious were found to be less willing to provide blood (M = 4.30, SD 0.74 vs. M = 4.49, SD 0.73; t(425) = 2.06, *p* = 0.04).

### 3.3. Data Management and Sharing

In the section on data management, a question investigated the level of agreement about the possibility of giving data access to eight persons or institutions. The three highest levels of agreement were to a general practitioner (M = 4.50; SD = 0.67), researchers (M = 4.15; SD = 0.81), and physicians in general (M = 3.94; SD = 1.04), followed by pharmaceutical companies (M = 2.49; SD = 1.12), the Italian state (M = 2.18; SD = 1.14), health insurance companies (M = 1.79; SD = 1.01), employers (M = 1.64; SD = 0.87), and lastly, by other for-profit insurance companies (M = 1.58; SD = 0.79).

A principal component analysis on these nine items resulted in an optimal two-factors solution after varimax rotation. These two components, both with eigenvalues greater than 1, accounted for 59% of the total variance. The first dimension comprised the five items related to allowing data access to institutions (pharmaceutical companies, the Italian state, health insurances, employers, and for-profit companies insurances; α = 0.81). The second dimensions comprised the items related to allowing data access to individuals working in the health sector (general practitioner, researchers, and physicians; α = 0.69). At the subgroup comparisons, composite scores showed that the younger (18–24) ground agreed more readily to give data to institutions (M = 2.34; SD = 0.71) compared to older respondents (M = 1.09, SD = 0.79; F(5,435) = 2.48, *p* = 0.031).

**Figure 1:**
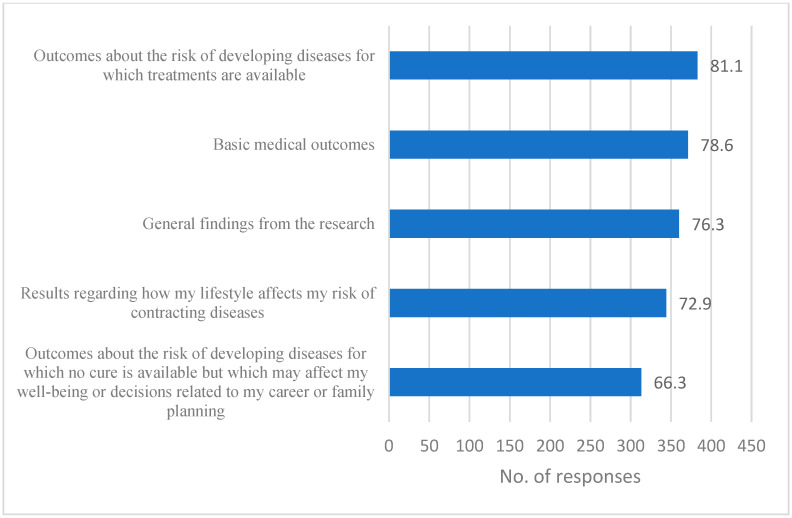
Results that respondents would like to receive.

A multiple-choice question asked which results respondents would like to receive concerning their health status in the case of participation in genomic research, and they could choose as many choices as relevant to them. The majority of the respondents expressed a strong interest in receiving the study results, with 81.1% of the respondents indicating a preference for information regarding their risk of developing a disease for which a cure is available, dropping to 66.3% when a cure is not available (Figure 1). In the subgroup comparisons via chi-square testing, the findings showed that the younger group (18–24) was significantly more interested in receiving basic medical results and information about curable diseases. Furthermore, individuals who perceive themselves as religious demonstrate a reduced level of receptivity to test results for diseases that currently have no possible treatment (33% vs. 25% of non-religious respondents; χ^2^ = 2.70, *p* = 0.061).

**Figure 2:**
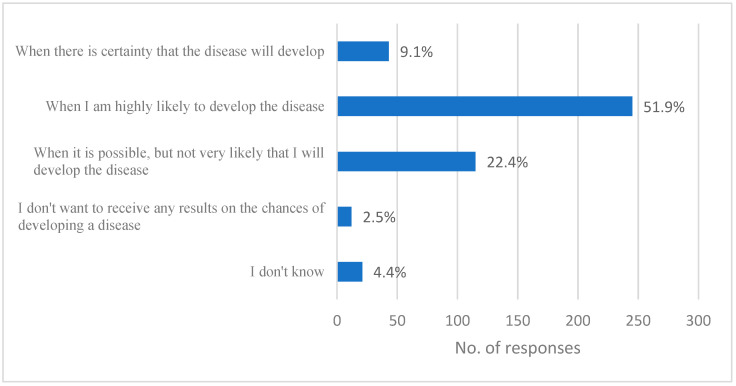
Minimum likelihood of developing a disease for which the respondent would like to be notified.

Finally, respondents were asked to indicate the minimum probability level at which respondents would like to receive results for a potential disease. Only 9.1% of respondents preferred to receive results when there was a certainty of developing a disease. In contrast, over half of the population (51.1%) preferred to receive results when the probability of developing the disease was high, and 22.4% preferred to receive results when the probability was possible but not very likely. Only 2.5% of the sample announced that would not be interested in receiving any results (Figure 2).

### 3.4. Concerns About Not Participating

The 35 respondents who said they would not participate in the genomic research study were asked about their reasons via nine Likert questions. Despite the small sample size, it is interesting to examine their concerns; however, due to the sample size and mild non-normality of the “Personal Disinterest” dimension, the results should not be considered as indicative. The three main concerns were about “Data and biological sample use” (M = 3.91; SD = 1.92), “Fear of data theft” (M = 3.69; SD = 1.47), and “Fear that companies may exploit the data and samples for profit” (M = 3.59; SD = 1.60). These were followed by “Fear that pharmaceutical companies have access to data and samples” (M = 3.53; SD = 1.58), “Concern about the research on own DNA” (M = 2.97; SD = 1.53), “Concern about discovering possible risks to my health” (M = 2.38; SD = 1.33), “Concern about DNA research in general” (M = 2.32; SD = 1.34), “No time to donate biological samples” (M = 1.79; SD = 1.25), and lastly, “No interest in health research” (M = 1.53; SD = 0.93).

A principal component analysis of the nine items resulted in an optimal three-factors solution after varimax rotation. These three components, all with eigenvalues greater than 1, accounted for 83% of the total variance. The first dimension comprised the four items related to “Concern about data privacy” (“Fear of data theft”, “Concern about data use”, “Fear that companies may exploit the data and biological samples for profit”, and “Fear that pharmaceutical companies have access to data and biological samples”; α = 0.90). The second dimension refers to “Personal disinterest” (“No interest in health research”, “No time to donate biological samples”, and “Concern about discovering possible risks to my health”; α = 0.93). The third dimension comprised items related to “Concern about DNA research” (“Concern about the research on my DNA” and “Concern about DNA research in general”; α = 0.92). The composite scores, all with excellent reliability, were used for subgroup comparisons. The “Concern related to data privacy” increased with age, being significantly higher in the two older age groups (18–24: M = 3.12.25, SD = 1.39; 25–34: M = 2.25, SD = 1.77; 35–44: M = 3.03, SD = 1.20, 45–54: M = 4.11, SD = 1.08, over 55: M = 4.72, SD = 0.36 (F(4,33) = 4.92, *p* = 0.004). Personal disinterest was instead higher in males (M = 2.05, SD = 1.48 vs. females M = 1.63, SD = 0.61; t(28) = 2.22, *p* = 0.027) and in alcohol consumers (M = 2.44, SD = 0.90 vs. M = 1.61, SD = 0.99; t(32) = 2.05, *p* = 0.017).

## 4. Discussion

This survey on public opinion about PM showed that a large majority of respondents (92.6%) indicated a willingness to participate in a genomic research project by providing health data and/or biological samples. This is a higher percentage than in previous studies conducted in Italy (86%) [30], Sweden (86%) [32], South Korea (83.5%) [27], Poland (65.3%) [25], Singapore (64%) [26], and Switzerland (53.6%) [23].

In the territory of the survey, a large genomic research project is currently underway. Among its objectives is the investigation of the genetic variability of the local population. The media coverage that has been given to the project in the region is likely a contributing factor with respect to the high percentage of participants who would be willing to participate. Additionally, in contrast to previous studies, the survey was conducted after the COVID-19 pandemic, an event that influenced the climate and perceptions towards healthcare [33]. A significant body of research has noted that patients were shown to be more comfortable sharing personal health information during the COVID-19 pandemic, particularly with care providers and researchers [34].

This ceiling effect is probably the reason for the absence of significant differences with respect to the percentages between the subgroups, i.e., those of different genders or religiosity or with a work background in the health sector; no association emerged with lifestyle variables either, although our results indicate a tendency toward lower willingness in younger respondents, comparable to the findings in previous studies [27,28].

Our study demonstrated that younger individuals with a higher level of education or prior work experience in the healthcare sector exhibited a greater awareness of PM, while other characteristics remained constant.

While the results indicate that the primary motivation for participating in scientific research is personal or familial benefit [25], notable variations are observed across different age groups. Participation in research for the benefit of society is significantly less important for younger people (18–24) than for other age groups, whilst people with only compulsory education had higher scores. In relation to the first aspect, it is noteworthy that the survey tends to confirm that young people in high-income Western societies are adopting individualistic social orientations [35], an increasing trend in recent decades [36]. A notable finding is that individuals with biological children exhibit a heightened sense of motivation to contribute to the benefit of both individuals and society, as well as a marginal inclination to disclose their family health history.

As has been shown in previous studies [23,24,25], the predominant concerns regarding participation pertain to data privacy, management, and dissemination. Individuals are less willing to share data derived from their social applications, as emerged in a previous study [23]. Young people are less likely to provide family medical/health history and data that are posted on social media. Despite the widespread use of social networks, a recent study has shown that younger people tend not to fully trust the information shared on them. It is therefore possible that this mistrust may make them more reluctant to share information posted online as they may believe it is not entirely reliable and therefore not usable for scientific health research [23]. Furthermore, it is noteworthy that younger individuals have more trust in institutions than other age groups, a phenomenon that has been documented by numerous agencies [37].

With regard to biological samples, individuals aged 18–24 are less willing to donate blood, feces, and tissue. Similarly, those aged 25–34 are also less willing to provide these samples, while the survey shows few differences with respect to gender: women are less willing to donate tissues, and respondents who prefer not to state their gender are also less willing to donate biological samples. Additionally, individuals who identify as religious are less likely to provide blood for research purposes. Limited research exists on the relationship between religiosity and blood donation, and the results are conflicting. Some authors have suggested a possible association between religiosity and blood donation [38], while others found a positive influence of religious beliefs on the attitude towards blood donation among youngsters in Iran [39]. In contrast, the findings of other studies have indicated an absence of a direct association between religiosity and blood donation [40,41].

The reasons why an individual may choose not to provide their biological data and samples are of significant interest. An analysis of the subgroup of respondents who were not willing to participate indicates that older age individuals are more concerned about data privacy issues than younger ones. While privacy and data protection concerns are not a novel topic in the extant literature [22,42], this finding introduces a new element of distinction. Previous studies on perceptions of online privacy did not reveal significant differences between older adults and younger individuals [43]. However, younger individuals belong to the “always connected” generation, having grown up with the internet and social media; thus, they tend to share posts and stories that document every aspect of their lives. This may lead to fewer concerns about privacy protection.

Finally, it is worth noting that men and regular alcohol consumers exhibited a lower level of interest in participating in the research. This finding aligns with the conclusions of previous studies, which indicated a general lack of interest among males in health-related subjects [44].

### Limitations

The present study is subject to several limitations. First, our convenience sample may be a limited representation of the population of the Aosta Valley; therefore, our findings have limited generalizability. While the final number of participants is large enough for a cross-sectional study, it cannot be excluded that individuals who are less likely to participate in genomic research projects involving the provision of their own biological samples have not even been willing to respond to an inquiry on the topic; it is necessary to consider a possible bias of the questionnaire responses. Furthermore, there is a relevant bias in the gender of respondents to the survey. The phenomenon of women being overrepresented in surveys on the availability of participation in health research projects is not a novel occurrence [24], and it can partially be attributed to the greater interest women tend to have in issues pertaining to health. In addition, lifestyle and health status showed no substantial influence. However, it should be noted that these data were self-reported, and future studies should consider using more objective measures. A further limitation is that while psychological and cultural factors are acknowledged to play a crucial role in shaping public perceptions of genomic research, our analysis primarily focused on demographic factors influencing awareness. Psychological and cultural dimensions have the potential to influence trust in scientific advancements, perceptions of risk, and willingness to participate. While these psychological and cultural factors were not the focus of this study, incorporating them in future research could provide a more comprehensive understanding of public attitudes toward genomic research.

Moreover, as highlighted in other studies [25], it is crucial to consider the potential for respondent fatigue in the process of answering. Despite efforts to minimize the number of questions and prepare them in a manner that is as understandable and accessible to respondents as possible, there is an ongoing risk of respondent fatigue leading to respondents completing the questionnaire in a hasty manner and answering questions in a manner that does not align with their genuine views. Furthermore, given the quantitative nature of the data collected in this study, no qualitative insights were obtained to explore concerns related to data privacy in the context of genomic research. Future studies should prioritize the collection of such data through focus groups and interviews. This approach would facilitate a comprehensive understanding of the participants’ concerns regarding data security. Moreover, it would provide policymakers with valuable information to encourage data donation.

Finally, another limitation is the lack of deeper examination into the relationship between awareness and knowledge of PM and willingness to participate. Further studies are necessary in order to investigate how to promote precision medicine literacy and to determine how increased awareness might influence participation.

## 5. Conclusions

PM research is gaining attention, and is at the center of health development strategies. This survey represents an important investigation following the implementation of a large-scale genomic research project in a designated area, exploring the willingness and concerns of participants regarding their involvement in the project.

This study showed that a large majority of the population is willing to provide data and biological samples to researchers. However, more in-depth analyses revealed that sociodemographic variables play a crucial role in the decision to participate. Our findings confirm that it is essential to consider these characteristics in the design phase of a genomic research project to foster engagement. For instance, communication campaigns targeting the youngest group, with the primary objective of educating them on the need to provide their biological data and samples for the benefit of society rather than for their own gain, and disseminating these messages through institutional channels rather than social networks, which are perceived as less trustworthy, could help to increase participation in genomic research and expand the databases available to researchers.

## Figures and Tables

**Table 1:** The percentage of respondents who indicated a willingness to participate in a genomic research project and who reported an awareness of PM.

	Willingness to Participate in a Genomic Study					Awareness of Precision Medicine				
		Yes	No				Yes	No		
	N (%)	N (%)	N (%)	χ^2^ (df)	*p*	N (%)	N (%)	N (%)	χ^2^ (df)	*p*
**Gender**				3.3 (2)	ns				2.7 (2)	ns
Females	328 (*69.5*)	306 (*93.3*)	22 (*6.7*)			328 (*69.5*)	117 (35.7)	211 (64.3)		
Males	128 (*27.1*)	118 (*92.2*)	10 (*7.8*)			128 (*27.1*)	54 (42.2)	74 (57.8)		
Prefer not to say or other	16 (*3.4*)	13 (*81.3*)	3 (*18.8*)			16 (*3.4*)	8 (*50.0*)	8 (*50.0*)		
**Age**				6.1 (6)	ns				11.3 (5)	**0.046**
18–24	48 (*10.2*)	42 (*87.5*)	6 (*12.5*)			48 (*10.2*)	9 (*18.8*)	39 (*81.3*)		
25–34	48 (*10.2*)	46 (*95.8*)	2 (*4.2*)			48 (*10.2*)	23 (*47.9*)	25 (*52.1*)		
35–44	100 (*21.2*)	90 (*90.0*)	10 (10.0)			100 (*21.2*)	37 (*37.0*)	63 (*63.0*)		
45–54	131 (*27.8*)	124 (*94.7*)	7 (*5.3*)			131 (*27.8*)	53 (*40.5*)	78 (*59.5*)		
55–64	123 (*26.1*)	113 (*91.9*)	10 (*8.1*)			123 (*26.1*)	46 (*37.4*)	77 (*62.6*)		
>64	22 (*4.7*)	22 (*100.0*)	0 (*0.0*)			22 (*4.7*)	11 (*50.0*)	11 (*50.0*)		
**Having biological children**				2.1 (1)	ns				1.4 (1)	ns
Yes	269 (*57.1*)	253 (*94.1*)	16 (*5.9*)			269 (*57.1*)	108 (*40.1*)	161 (*59.9*)		
No	201 (*42.8*)	182 (*90.5*)	19 (*9.5*)			201 (*42.8*)	70 (*34.8*)	131 (*65.2*)		
**Education**				0.3 (3)	ns				23.9 (3)	**0.001**
Compulsory education	26 (*5.5*)	24 (*92.3*)	2 (*7.7*)			26 (*5.5*)	6 (*23.1*)	20 (*76.9*)		
Secondary	186 (*39.4*)	172 (*92.5*)	14 (*7.5*)			186 (*39.4*)	49 (*26.3*)	137 (*73.7*)		
Tertiary	205 (*43.4*)	191 (*93.2*)	14 (*6.8*)			205 (*43.4*)	96 (*46.8*)	109 (*53.2*)		
Post-university degree	55 (*11.7*)	50 (90.9)	5 (9.1)			55 (*11.7*)	28 (*50.9*)	27 (*49.1*)		
**Work experience in the healthcare sector**				0.1 (1)	ns				7.9 (1)	**0.005**
Yes	59 (*12.6*)	54 (*91.5*)	*5 (*8.5*)*			59 (*12.6*)	32 (*54.2*)	27 (*45.8*)		
No	411 (*87.4*)	381 (*92.7*)	30 (*7.3*)			411 (*87.4*)	145 (*35.3*)	266 (*64.7*)		
**Alcohol consumptions**				0.1 (1)	ns				0.2 (1)	ns
Yes/More than twice a week	152 (*32.2*)	140 (*92.1)*	12 (*7.9*)			152 (*32.2*)	60 (*39.5*)	92 (*60.5*)		
No/Less than twice a week	319 (*67.6*)	296 (*92.8*)	23 (*7.2*)			319 (*67.6*)	118 (*37.0*)	201 (*63.0*)		
**Smoker**				3.1 (1)	ns				2.3 (1)	ns
Yes	62 (*13.1*)	54 (*87.1*)	8 (*12.9*)			62 (*13.1*)	18 (*29.0*)	44 (*71.0*)		
No	409 (*86.7*)	382 (*93.4*)	27 (*6.6*)			409 (*86.7*)	160 (*39.1*)	249 (*60.9*)		
**Health self-perceived status**				0.9 (2)	ns				2.8 (2)	ns
In health	56 (*11.9*)	53 (*94.6*)	3 (*5.4*)			56 (*11.9*)	26 (*46.4*)	30 (*53.6*)		
Good Health	364 (*77.1*)	337 (*92.6*)	27 (*7.4*)			364 (*77.1*)	131 (*36.0*)	233 (*64.0*)		
Excellent health	49 (*10.4*)	44 (*89.8*)	5 (*10.2*)			49 (*10.4*)	28 (*57.1*)	21 (*42.9*)		

Bold: Statistical Significance; Italics: Percentage; ns: statistically non-significant.

## Data Availability

The raw data supporting the conclusions of this article will be made available by the authors on request.

## Supplementary Materials

The following supporting information can be downloaded at: https://www.mdpi.com/article/10.3390/jpm15030080/s1, Questionnaire, English version.
