# Supplementary material for: The Public Knowledge of Precision Medicine and Genomic Research: A Survey in the Aosta Valley
Source: J Pers Med. 2025 Feb 24;15(3):80. doi: 10.3390/jpm15030080 (PMC11943031; doi:10.3390/jpm15030080)
Supplement: Supplementary file 1 [file jpm-15-00080-s001.zip › jpm-3390326-supplementary.pdf]

Survey on Personalized Medicine

Welcome!

This is our questionnaire on personalized medicine. Completing the questionnaire will take only 10 minutes, and your answers will be collected anonymously.

What is personalized medicine?

It is an *"emerging approach for disease treatment and prevention that takes into account individual genetic variables, environmental factors, and lifestyle."* Please answer the questionnaire truthfully.

Privacy and Terms of Use

Dear User,

This survey is provided by the University of Valle d'Aosta. The main goal is to understand how people feel about precision medicine and whether they would be willing to undergo genetic testing as part of the 5000Genomi Research Project. The data will be collected and then analyzed as a whole and in a way that cannot identify any individual. This will be done in order to conduct statistical research. The University needs to process the data from the questionnaire to carry out the research project. This is necessary for a task that helps the public or is connected to the University's public authority (legal basis: Article 6 (1) (e) GDPR).Participation in the project and completing the questionnaire are voluntary. The data collected will be processed electronically and in compliance with the technical and organizational security measures required for the processing of personal data. The answers provided will be stored for the time necessary for statistical analysis and the preparation of final reports. For more information or to exercise the rights provided by the GDPR (Articles 15 to 22), you can send an email to **info@univda.it** or contact the Data Protection Officer at **rpd@univda.it**.

Part 1: Consent to Participate in the Research

1. **Do you agree to participate in the research?**

☐ Yes, I consent
☐ No, I do not consent

Part 2: General Perspective

2. Have you ever heard of "personalized medicine" before today?

- ☐ Yes
- ☐ No *(skip to question 5)*

3. How did you learn about it? *(Select all that apply)*

- ☐ Newspapers
- ☐ Scientific journals
- ☐ Television
- ☐ Social media
- ☐ Friends and/or Acquaintances
- ☐ Doctor
- ☐ Other

4. What are the first three or more words that come to your mind when thinking about "personalized medicine"? *(Open-ended response)*

5. How important are the following aspects in deciding whether to participate in a medical-scientific study?

	**Not important**	**Slightly important**	**Moderately important**	**Very important**	**Extremely important**
The research could improve people's health	☐	☐	☐	☐	☐
Receiving money in exchange for participation	☐	☐	☐	☐	☐
Getting information about my health	☐	☐	☐	☐	☐

6. Would you participate in a medical research project that uses your health data and/or biological samples?

- ☐ Yes *(skip to question 8)*
- ☐ No *(skip to question 7)*

7. What would be your main reasons for NOT participating in medical research? (then, go to question 21)

	Strongly disagree	Disagree	Neutral	Agree	Strongly agree
I am concerned about DNA research in general	☐	☐	☐	☐	☐
I worry about research on my own DNA	☐	☐	☐	☐	☐
I am afraid of discovering possible health risks	☐	☐	☐	☐	☐
I am concerned about the use of my data and samples	☐	☐	☐	☐	☐
I fear data and sample theft	☐	☐	☐	☐	☐
I worry that pharmaceutical companies may access my data and samples	☐	☐	☐	☐	☐
I fear companies may exploit my data and samples for profit	☐	☐	☐	☐	☐
I am afraid of needles and procedures required to donate samples	☐	☐	☐	☐	☐
I am not interested in health research	☐	☐	☐	☐	☐
I don't have time to donate data and samples	☐	☐	☐	☐	☐

8. What would be your main reasons for PARTICIPATING in medical research?

	Strongly disagree	Disagree	Neutral	Agree	Strongly agree
To advance scientific knowledge	☐	☐	☐	☐	☐
To benefit society	☐	☐	☐	☐	☐
Sense of duty	☐	☐	☐	☐	☐
To improve healthcare	☐	☐	☐	☐	☐
To learn something about my health	☐	☐	☐	☐	☐

	Strongly disagree	Disagree	Neutral	Agree	Strongly agree
To benefit my family (e.g., knowing genetic predispositions)	☐	☐	☐	☐	☐

9. How important would it be to provide health data and biological samples for medical research?

	Not important at all	Slightly important	Moderately important	Very important	Extremely important
For myself	☐	☐	☐	☐	☐
For my family	☐	☐	☐	☐	☐
For society	☐	☐	☐	☐	☐

10. How willing would you be to share the following personal data for medical research?

	Strongly disagree	Disagree	Neutral	Agree	Strongly agree
Information about my health status	☐	☐	☐	☐	☐
My medical records	☐	☐	☐	☐	☐
My family's medical history	☐	☐	☐	☐	☐
My social media data	☐	☐	☐	☐	☐
My data from mobile health apps (e.g., step counter, calorie tracker)	☐	☐	☐	☐	☐

11. How willing would you be to provide the following biological samples for medical research?

	Strongly disagree	Disagree	Neutral	Agree	Strongly agree
Blood	☐	☐	☐	☐	☐
Saliva	☐	☐	☐	☐	☐

	**Strongly disagree**	**Disagree**	**Neutral**	**Agree**	**Strongly agree**
Urine	☐	☐	☐	☐	☐
Feces	☐	☐	☐	☐	☐
Hair	☐	☐	☐	☐	☐
Tissues	☐	☐	☐	☐	☐

Part 3: Data Management and Sharing

12. The storage of biological samples for research purposes is undertaken in specialised biobanks. These facilities are designed to collect and store health data and biological samples for utilisation in biomedical research.How important would it be for you to know the following information before donating your data and/or biological samples to a biobank?

	Not important	Slightly important	Moderately important	Very important	Extremely important
Potential risks and benefits of donation	☐	☐	☐	☐	☐
How my data and samples will be stored	☐	☐	☐	☐	☐
Who will have access to my data and samples	☐	☐	☐	☐	☐
Who will benefit from the research	☐	☐	☐	☐	☐
Who will financially profit from the research	☐	☐	☐	☐	☐
Security measures for data and sample protection	☐	☐	☐	☐	☐

13. Who do you think should own the data and samples donated to a biobank? *(Select all that apply)*

- ☐ Myself
- ☐ The biobank
- ☐ The Italian government
- ☐ Universities involved in the research with the biobank
- ☐ Researchers who make discoveries
- ☐ No one
- ☐ Others
- ☐ I don't know

14. In your opinion, who should be responsible for the proper storage and management of your data and samples? *(Select all that apply)*

- ☐ The biobank or its board of directors
- ☐ An independent committee representing citizens

☐ An independent expert committee (e.g., independent researchers, scientists, and clinicians not associated with the biobank)
☐ A mixed committee of experts and non-experts
☐ The Italian government (e.g., the Ministry of Health or Research)
☐ The research sponsor
☐ Other

15. Would you agree to allow the following individuals and/or institutions access to your data and samples?

	Strongly disagree	Disagree	Neutral	Agree	Strongly agree
My primary care doctor	☐	☐	☐	☐	☐
Doctors in general	☐	☐	☐	☐	☐
University researchers	☐	☐	☐	☐	☐
Pharmaceutical companies	☐	☐	☐	☐	☐
Other for-profit companies	☐	☐	☐	☐	☐
Health insurance companies	☐	☐	☐	☐	☐
My employers	☐	☐	☐	☐	☐
The Italian Government	☐	☐	☐	☐	☐

16. How often would you like the biobank to ask for your permission to use your data and/or samples in future research projects? *(Keep in mind that your decision to provide data can always be changed or withdrawn.)*

☐ Only once, at the time I provide my data or samples
☐ Every time my data or samples are needed for a new project
☐ It depends on the type of project
☐ I don't know

17. In what form would you like your data and samples to be stored?

☐ **Anonymized** – Data cannot be linked to a specific person. Privacy risks are low since it is nearly impossible to trace the data back to the individual.
☐ **Pseudonymized** – Personal data is linked to a specific person through a securely stored code. The risk is limited, as data can only be identified with the code.
☐ **Identifiable** – Data is stored and labeled with a name. Privacy risks are higher since it is easy to identify the data.

Part 4: Receiving Personal Health Results

18. If you participated in research involving your health data and samples stored in a biobank, what types of results would you like to receive? *(Select all that apply)*

☐ Basic medical results (e.g., lab tests)
☐ Information on how my lifestyle affects my disease risk (e.g., smoking, obesity)
☐ Risk of developing diseases for which treatments are available
☐ Risk of developing diseases without available treatments but that could impact my well-being, career, or family planning
☐ General findings from the research

19. Often, results related to disease development can only be expressed in terms of probability. What level of risk of developing a disease would be sufficient for you to want to see the result?

☐ When there is certainty that I will develop the disease
☐ When it is highly probable that I will develop the disease
☐ When there is a chance, but it is not very likely, that I will develop the disease
☐ I would not want to receive any results regarding the probability of developing a disease
☐ I don't know

20. If you wanted to receive some or all of the research results, through which communication channel would you prefer to receive them? *(Select all that apply)*

☐ By letter via postal mail
☐ By phone call
☐ By email
☐ On a website with secure login access
☐ Through a mobile app
☐ Through face-to-face communication
☐ I don't know

Part 5: E-Health: Health Protection and Digital Tools

21. Do you know what Artificial Intelligence is?

- ☐ Yes
- ☐ No *(skip to question 23)*

22. What are the first three or more words that come to mind to describe the use of Artificial Intelligence in the healthcare sector? *(Open-ended response)*

23. Do you use wearable devices (e.g., smartwatches, fitness trackers) that record health and lifestyle data?

- ☐ Yes
- ☐ No *(skip to question 25)*

24. How often do you use wearable devices?

- ☐ Regularly (every day)
- ☐ Often (5-6 times a week)
- ☐ Sometimes (3-4 times a week)
- ☐ Rarely (1-2 times a week)

Part 6: Personal Information

25. What is your gender?

- ☐ Male
- ☐ Female
- ☐ Other
- ☐ Prefer not to say

26. What is your nationality?

- ☐ Italian
- ☐ Other

27. Do you live in Valle d'Aosta?

- ☐ Yes
- ☐ No

28. How old are you?

- ☐ 18-24
- ☐ 25-34
- ☐ 35-44
- ☐ 45-54
- ☐ 55-64
- ☐ 65-74
- ☐ 75 or older

29. What is your highest level of education?

- ☐ Primary school diploma
- ☐ Middle school diploma
- ☐ Professional qualification (three-year vocational school)
- ☐ High school diploma
- ☐ Bachelor's degree
- ☐ Master’s degree / Single-cycle degree
- ☐ First or Second level Master’s degree
- ☐ PhD
- ☐ None of the above

30. Are you currently enrolled in a university program?

- ☐ Yes
- ☐ No

31. Do you have biological children?

- ☐ Yes
- ☐ No

32. Do you smoke?

- ☐ Yes
- ☐ No *(skip to question 34)*

33. How frequently do you smoke?

- ☐ Less than 1 cigarette per day
- ☐ Between 1 and 5 cigarettes per day
- ☐ Between 5 and 10 cigarettes per day
- ☐ Between 10 and 15 cigarettes per day
- ☐ More than 15 cigarettes per day

34. How often do you consume alcoholic beverages (in quantities exceeding a glass of wine or a small beer)?

- ☐ I am a teetotaler
- ☐ Less than once a week
- ☐ 1 to 2 times a week
- ☐ 3 to 4 times a week
- ☐ 5 or more times a week

35. Do you consider yourself a sporty person?

- ☐ Yes
- ☐ No *(skip to question 37)*

36. How many days a week do you practice sports?

- ☐ Occasionally (1-2 times a week)
- ☐ Often (3-4 times a week)
- ☐ Regularly (5-6 times a week)
- ☐ Every day

37. How would you describe your health status?

- ☐ In very poor health
- ☐ In poor health
- ☐ In good health
- ☐ In excellent health

38. Would you describe yourself as a religious person?

- ☐ Yes
- ☐ No

39. What is your monthly net salary?

- ☐ Less than €800
- ☐ Between €801 and €1500
- ☐ Between €1501 and €2200
- ☐ Between €2201 and €3000
- ☐ Between €3001 and €5000
- ☐ More than €5000

40. Do you currently work or have you ever worked in the healthcare sector (hospitals, nursing homes, dental offices, etc.)?

- ☐ Yes
- ☐ No

41. Have you ever heard of the 5000Genomi@VdA Project?

- ☐ Yes
- ☐ No

Thank you for your participation!
